# FoxO Proteins in the Nervous System

**DOI:** 10.1155/2015/569392

**Published:** 2015-06-10

**Authors:** Kenneth Maiese

**Affiliations:** Cellular and Molecular Signaling, Newark, NJ 07101, USA

## Abstract

Acute as well as chronic disorders of the nervous system lead to significant morbidity and mortality for millions of individuals globally. Given the ability to govern stem cell proliferation and differentiated cell survival, mammalian forkhead transcription factors of the forkhead box class O (FoxO) are increasingly being identified as potential targets for disorders of the nervous system, such as Alzheimer's disease, Parkinson's disease, Huntington's disease, amyotrophic lateral sclerosis, and auditory neuronal disease. FoxO proteins are present throughout the body, but they are selectively expressed in the nervous system and have diverse biological functions. The forkhead O class transcription factors interface with an array of signal transduction pathways that include protein kinase B (Akt), serum- and glucocorticoid-inducible protein kinase (SgK), I*κ*B kinase (IKK), silent mating type information regulation 2 homolog 1 (*S. cerevisiae*) (SIRT1), growth factors, and Wnt signaling that can determine the activity and integrity of FoxO proteins. Ultimately, there exists a complex interplay between FoxO proteins and their signal transduction pathways that can significantly impact programmed cell death pathways of apoptosis and autophagy as well as the development of clinical strategies for the treatment of neurodegenerative disorders.

## 1. Clinical Significance of Nervous System Disorders

Nervous system disorders lead to disability and death in a significant proportion of the world's population. For example, almost ten percent of the global population suffers from the sporadic form of Alzheimer's disease (AD) while familial cases of AD account for less than 2% of all presentation [1, 2]. In the United States alone, greater than 5 million individuals have AD and another 3.5 million individuals are under treatment at an annual cost of almost 4 billion US dollars. In regards to cerebrovascular disease, stroke is presently ranked as the fourth leading cause of death and can also affect the lives of millions of individuals [3]. A number of factors are responsible for stroke no longer being ranked higher as a cause of death. These factors include improved management of hypertension and diabetes, reduction in tobacco consumption, heightened public awareness for seeking rapid care [3, 4], treatment with recombinant tissue plasminogen activator [5], and novel new strategies that focus on trophic factors, improved biomarkers, and cellular pathways of oxidative stress [3, 6–10].

Yet, the availability of treatments that can prevent the initiation of acute or chronic neurodegenerative disorders or block the progression of these diseases is scarce. Therapeutic strategies that can aggressively treat AD and stroke continue to remain limited for most individuals. Furthermore, multiple other neurodegenerative disorders also greatly impact the global population with treatments that are not always optimal. By the year 2030, epilepsy is predicted to affect over 50 million people, neuropathies are estimated to afflict almost 300 million individuals, and neurological injuries may alter the lives of 243 million individuals [11].

## 2. Targeting Forkhead Transcription Factors

Given the need for novel directions that can potentially diminish or resolve the onset and progression of neurological disorders, mammalian forkhead transcription factors are surfacing as potential effective targets that can offer new developments for drug discovery. Since the documentation of the* Drosophila melanogaster gene forkhead*, greater than 100 forkhead genes, and 19 human subgroups that range from* FOXA* to* FOXS* is now known to exist [12]. Prior terminology for forkhead proteins included forkhead in rhabdomyosarcoma (FKHR) (FOXO1), FKHRL1 (forkhead in rhabdomyosarcoma like protein 1) (FOXO3a), the* Drosophila* gene fork head (*fkh*), Forkhead Related Activator- (FREAC-) 1 and FREAC-2, and the acute leukemia fusion gene located in chromosome X (*AFX*) (*FOXO4*) [13, 14]. For the current nomenclature, an Arabic number is provided with the designation of “Fox,” and then a subclass or subgroup letter is provided, and finally the member number is listed within the subclasses of the Fox proteins [15]. All letters are capitalized for human Fox proteins. For the mouse, only the initial letter is listed as uppercase and for all other chordates the initial and subclass letters are uppercased [16–19].

Mammalian FOXO proteins are assigned to the O class of the forkhead box class transcription factors and consist of FOXO1, FOXO3, FOXO4, and FOXO6 [20]. With a butterfly-like appearance on X-ray crystallography [21] and nuclear magnetic resonance [22], the forkhead box (FOX) family of genes has a conserved forkhead domain (the “forkhead box”) described as a “winged helix.” The forkhead domain in FoxO proteins has three *α*-helices, three *β*-sheets, and two loops that compose the “wings” of the domain [23] which is specific for the forkhead proteins, since not all winged helix domains are considered to be Fox proteins [24]. The *α*-helices and *β*-sheets have high sequence homology with variations in either absent *β*-sheets and loops or additional *α*-helices. As transcription factors, FoxO proteins bind DNA through the FoxO-recognized element in the C-terminal basic region of the forkhead DNA binding domain [25, 26]. Target gene expression is repressed or activated through fourteen protein-DNA contacts with the primary recognition site located at *α*-helix H3 [21]. Phosphorylation or acetylation that can block FoxO activity may alter the binding of the C-terminal basic region to DNA to prevent transcriptional activity [27]. However, multiple mechanisms may contribute to forkhead DNA binding that involve variations in the N-terminal region of the recognition helix, changes in electrostatic distribution, and nuclear translocation of FoxO proteins [28–31].

FoxO proteins are expressed in all tissues of the body (Table 1). In relation to metabolic signaling, function of FoxO proteins appears to be conserved among multiple species that include* Caenorhabditis elegans, Drosophila melanogaster,* and mammals. FoxO proteins are homologous to the transcription factor DAuer Formation-16 (DAF-16) in the worm* Caenorhabditis elegans* that can determine metabolic insulin signaling and lead to lifespan extension [32, 33]. Furthermore, individual FoxO proteins appear to have selective expression in the nervous system that may provide clues to the biology for specific FoxO proteins [26, 34]. FoxO6 may oversee memory consolidation and emotion [35], since it is present in several regions of the brain, such as the hippocampus, the amygdala, and the nucleus accumbens [36, 37]. FoxO1 may have a vital role in a number of functions given its broad expression that may relate to astrocyte survival [38], modulation of embryonic endothelial stem cell survival [39], regulation of ischemic brain injury [10], vascular disease [40], and motor and memory pathways in the striatum and subregions of the hippocampus [36]. FoxO3 may have a more critical role in auditory synaptic transmission [41], cerebral endothelial vascular cell survival [42, 43], oxidative stress injury in mouse cerebellar granule neurons [44], neonatal hypoxic-ischemic encephalopathy [45], erythroid cell growth [46], and hippocampal neuronal injury [47, 48].

## 3. Epigenetic and Posttranslation Modification of Forkhead Transcription Factors

Activity of FoxO proteins is controlled by epigenetic [44, 49] and posttranslation protein modifications that involve phosphorylation [28, 30, 46–48, 50–56], acetylation [44, 50, 57], and ubiquitylation [26, 58–60] of these proteins (Table 1). Phosphorylation of forkhead transcription factors can be mediated by the serine-threonine kinase protein kinase B (Akt) [2, 61–66]. In the nervous system, Akt can protect cells during ischemic preconditioning [67], beta-amyloid (A*β*) toxicity [68–70], oxidative stress injury in the retina [71], inflammatory vascular injury [72], cerebral ischemia [73], experimental subarachnoid hemorrhage [74], flavonoid-dependent neuroprotection [75], lipoic acid protection [76, 77], epidermal growth factor receptor transactivation [78], neuroinflammation [79], tau homeostasis [80], senile plaque memory impairment [81], and growth factor administration [28, 71, 82–89]. Akt phosphorylates FoxO proteins that will bind FoxOs to 14-3-3 proteins prevent nuclear translocation and block the transcription of target genes that promote apoptosis [47, 52, 90, 91]. Akt also may control FoxO proteins activity and subsequently block caspase cleavage to prevent the induction of apoptotic cell death. Akt suppresses caspase activity that ultimately leads to mitochondrial pore opening and cytochrome c release [42, 66, 92–101]. Enhanced activity of FoxO proteins such as FoxO3a also can lead to cytochrome c release and caspase-induced apoptotic death [28, 51, 57, 66, 102–104]. As a result, one mechanism by which Akt prevents apoptotic cell death is through the blockade of FoxO protein activity that would prevent caspase activation. In addition, pathways such as Akt that block caspase 3 activity appear to offer another unique regulatory mechanism. Caspase 3 cleavage of FoxO3a may result in “proapoptotic” amino-terminal (Nt) fragments that can lead to cell death [105]. However, during caspase 3 inhibition such as that by Akt, phosphorylated FoxO3a remains intact and does not lead to apoptotic cell injury during oxidative stress [53, 106].

In addition to Akt, other pathways can lead to the phosphorylation and inactivation of FoxO proteins. The serum- and glucocorticoid-inducible protein kinase (SgK), a member of a family of kinases termed AGC (protein kinase A/protein kinase G/protein kinase C) kinases that includes Akt and phosphorylates FoxO3a and maintains this protein in the cytoplasm [107]. Importantly, Akt and SgK can phosphorylate FoxO proteins at different sites, suggesting greater options to control FoxO protein activity. However, some protein kinases such as mammalian sterile 20-like kinase-1 (MST1) can phosphorylate FOXO proteins and disrupt the binding to 14-3-3 which then allows FOXO nuclear translocation and subsequent death in neurons [29], indicating that the phosphorylation site of FoxO proteins is crucial in determining the activity of forkhead transcription factors. The ability of MST1 to activate FoxO proteins may be linked to c-Jun N-terminal kinase (JNK), since MST1 can increase JNK activation [108] which phosphorylates 14-3-3 protein, blocks binding to FoxO, and results in the nuclear localization of FoxO proteins [109].

Pathways associated with ubiquitylation and acetylation also control posttranslational modification of FoxO proteins [110, 111]. For example, Akt also leads to the ubiquitination and degradation through the 26S proteasome of FoxO proteins [111, 112]. Agents that can prevent the ubiquitination and degradation of FoxO proteins may serve as important agents to induce apoptotic cell death in cancers that can be tied to silent mating type information regulation 2 homolog 1 (*S. cerevisiae*) (SIRT1) [50, 113]. In a similar vein, SIRT1 activity also can lead to enhanced cell survival such as in the nervous system through inhibition of FoxO activity [57, 114–117]. Mammalian forkhead transcription factors can bind to the SIRT1 promoter region that contains a cluster of five putative core binding repeat motifs (IRS-1) and a forkhead-like consensus-binding site (FKHD-L) to affect FoxO transcription [118]. FoxO proteins, such as FoxO1, can subsequently regulate SIRT1 transcription and increase SIRT1 expression [119]. In some cases, SIRT1 and FoxO proteins may function synergistically to promote cell survival. In differentiated chondrocytes exposed to oxidative stress, loss of the forkhead transcription factors FoxO1 and FoxO3 in combination with decreased SIRT1 activity lead to cell death with reduced production of autophagic related proteins, indicating that SIRT1 with FoxO proteins may be necessary for cellular survival [120]. I*κ*B kinase (IKK) also can directly phosphorylate and block the activity of FoxO proteins that results in the proteolysis of FoxO3a via the Ub-dependent proteasome pathway [121]. Acetylation of FoxO proteins provides another avenue for the control of these proteins. FoxO proteins are acetylated by histone acetyltransferases that include p300, the CREB-binding protein (CBP), and the CBP-associated factor. Once acetylated such as CBP, FoxO proteins translocate to the cell nucleus but have diminished activity since acetylation of lysine residues on FoxO proteins has been shown to limit the ability of FoxO proteins to bind to DNA [122]. Furthermore, acetylation can increase phosphorylation of FoxO proteins through Akt [122]. FoxO proteins are deacetylated by histone deacetylases, such as SIRT1 [13, 112, 123, 124]. Histone deacetylase 2 (HDAC2) also forms a physical complex with FoxO3a. This complex can influence FoxO3a-dependent gene transcription and oxidative stress-induced mouse cerebellar granule neuron cell death [44].

## 4. Forkhead Transcription Factors, Oxidative Stress, Apoptosis, and Autophagy

FoxO proteins are important components in the control of cell survival and neurodegenerative disorders determined by apoptosis and autophagy in the presence of oxidative stress [7, 125–128]. During oxidative stress, reactive oxygen species (ROS) are generated that include nitric oxide, peroxynitrite, superoxide free radicals, hydrogen peroxide, and singlet oxygen [97, 129–135]. These ROS can lead to cellular organelle injury, protein misfolding, DNA destruction, and neuronal synaptic dysfunction [48, 132, 136–138]. Endogenous systems exist in the body to prevent cellular injury during oxidative stress, but these systems can become overwhelmed such as glutathione peroxidase [139, 140], superoxide dismutase [120, 132, 138, 141–148], and vitamins B, C, D, and K [59, 140, 149–151]. FoxO proteins have been linked to disease progression and oxidative stress such as that with vitiligo [134] (Table 1). In patients with polymorphism of the* FOXO3A* gene, FOXO3A levels and catalase enzyme activity in vitiligo patients were decreased compared with control groups, suggesting in this case that FoxO proteins may confer protection. In other systems such as the maternal decidua, FoxO proteins may function independently in regards to oxidative stress with FOXO1 preventing oxidative stress damage and FOXO3a promoting oxidative cell death [152]. In addition, oxidative stress can serve as an epigenetic modifier of FoxO interactions with other proteins that can influence neuronal cell survival [44].

Autophagy is a process that recycles cytoplasmic components while removing dysfunctional organelles for tissue remodeling [7, 153–156]. Macroautophagy is the most prevalent type of autophagy that sequesters cytoplasmic proteins and organelles into autophagosomes [6, 157–160] and plays a role with FoxO proteins [2, 49, 127]. Autophagosomes, once produced, combine with lysosomes for degradation and are recycled for future cellular processes [125, 159, 161–163]. Under conditions of oxidative stress, FoxO proteins can lead to the induction of autophagy and promote cell survival (Table 1). During exposure with the oxidant tert-butyl hydroperoxide, constitutive active form of FoxO3 increases human articular chondrocyte cell viability and the expression of autophagy related proteins [120]. SIRT1-mediated deacetylation of FoxO1 also appears to mediate starvation-induced increases in autophagic flux that can maintain left ventricular function during periods of starvation [164]. Cardiac expression of constitutively active FoxO3 results in reversible heart atrophy through the activation of autophagic pathways [165]. In experimental models of full-length mutant Huntingtin (mHtt) transgenic mice, ectopic expression of FoxO1 enhances autophagy and toxic mHtt protein clearance in neuronal cell cultures [160]. However, under some conditions, a reduction in autophagy has been reported in the presence of increased FoxO expression, suggesting that FoxO cytoprotection may not always be directly tied to the induction of autophagy. Upregulation of FoxO3 and SIRT1 with a reduction in autophagy occurs in human bronchial epithelial cells exposed to cigarette smoke condensates in the presence of Amurensis H (Vam3), a dimeric derivative of resveratrol that can reduce oxidative stress [166].

In regards to the programmed cell death pathway of apoptosis, a later phase that leads to genomic DNA degradation is preceded by an early phase with the loss of plasma membrane lipid phosphatidylserine (PS) asymmetry [156, 167, 168]. The later phase of apoptosis results in DNA destruction [8, 19, 169–171], but the early phase of apoptosis represents an important target to save injured cells. Prevention or reversal of membrane PS externalization [68, 172–177] can result in the salvage of neurons and prevent inflammatory cells such as microglia from removing otherwise functional neurons [174, 178, 179]. During oxidative stress, FoxO proteins can lead to initial membrane PS externalization and subsequent DNA degradation (Table 1). In the presence of high glucose exposure, the development of endothelial cell dysfunction occurs with a reduction in SIRT1 expression and an increase in FoxO1 expression [180]. It has been suggested that FoxO proteins, such as FoxO1 and FoxO3a, must be present for oxidative stress to result in apoptosis [181]. This observation is supported by cell culture and animal studies demonstrating that inhibition or gene knockdown of FoxO1 or FoxO3a results in stroke reduction by estradiol [91], protects against microglial cell demise during oxidative stress [106] and A*β* exposure [182], promotes the protective effects of metabotropic glutamate receptors [102], increases neuronal cell survival through nicotinamide adenine dinucleotide (NAD^+^) precursors [51], and provides trophic factor protection with erythropoietin (EPO) [28, 42, 46, 52] and neurotrophins [183–185]. However, under some scenarios that may impact other cellular signal transduction pathways, the activation of FoxO proteins may prevent apoptotic cell injury during oxidative stress such as chondrocytes [120]. Other studies show that in some cellular populations such as mouse hematopoietic stem cells, the conditional deletion of FoxO1, FoxO3a, and FoxO4 can lead to an increase in ROS [186], suggesting that FoxO proteins may be beneficial in regulation ROS in some cellular environments.

FoxO proteins such as FoxO3a can lead to the induction of “proapoptotic genes” and disrupt proliferative pathways of Wnt signaling [50]. A converse relationship exists between Wnt signaling and FoxO proteins. For example, FoxO3a can block prostate cell malignant phenotypes through the downregulation of Wnt signaling and *β*-catenin [187]. Wnt signaling includes the family member Wnt1 that can oversee neuronal development, angiogenesis, immunity, tumorigenesis, and stem cell proliferation [188–192]. Wnt1 expression is increased during injury of endothelial cells [28], metabolic disturbance [28], nonneuronal cell activation [69, 104, 193–195], spinal cord injury [196], stroke [197], and oxidative stress [104, 179, 197]. This increased expression of Wnt1 appears to be protective since loss of Wnt1 translates into progressive spinal cord injury [198], impaired neurogenesis [199], and apoptosis [156, 193, 200]. Wnt1 signaling pathways can prevent cellular injury during experimental diabetes [28, 201], ischemic brain injury [197, 202], dopaminergic neuronal cell injury [179, 189, 195, 203], toxic environments for microglia and other inflammatory cells [69, 104, 191, 193], and neuronal synaptic dysfunction [204]. Wnt signaling can afford cellular protection against apoptotic cell death through the inactivation of FoxO proteins. Phosphorylation and inhibition of FoxO3a activity by *β*-catenin during oxidative stress can protect hepatocytes from apoptotic cell death [54]. Osteoblastic differentiation can be preserved in the presence of oxidative stress through the increased expression of Wnt signaling pathways and the inhibition of FoxO3a [205]. In microglial cells of the central nervous system, Wnt1 prevents apoptosis through the posttranslational phosphorylation and maintenance of FoxO3a in the cytoplasm to prevent the loss of mitochondrial membrane permeability, cytochrome c release, Bad phosphorylation, and activation of caspases [104]. Neuroprotective trophic factors and cytokines, such as EPO [83, 87, 206, 207], also use Wnt signaling to offer cellular protection through the inhibition of FoxO proteins. EPO protects cerebral endothelial cells during oxygen-glucose deprivation by phosphorylating FoxO3a and preventing its subcellular trafficking to the nucleus [42, 208]. During elevated glucose exposure, EPO relies upon Wnt1 to block FoxO3a activity and maintain cerebral endothelial survival [28]. Wnt1 inducible signaling pathway protein 1 (WISP1), also known as CCN4, is a target of Wnt1 and affects programmed cell death, cancer cell growth, extracellular matrix production, cellular migration, and mitosis [159, 209–213]. WISP1 also protects neurons through the posttranslational phosphorylation of FoxO3a, by sequestering FoxO3a in the cytoplasm with protein 14-3-3, and by limiting deacetylation of FoxO3a [47]. Overexpression of FoxO3a during oxidative stress results in caspase 1 and caspase 3 [58, 214]. Through an autoregulatory loop, WISP1 has been shown to increase neuronal survival by limiting FoxO3a deacytelation, blocking caspases 1 and 3 activation, and fostering SIRT1 nuclear trafficking [47]. It should be noted that, under some conditions, Wnt signaling through *β*-catenin may increase* FoxO* transcriptional activity and competitively limit *β*-catenin interaction with members of the lymphoid enhancer factor/T cell factor family [215].

## 5. Forkhead Transcription Factors, Development, Stem Cell Proliferation, and Neurodegeneration

FoxO proteins have a prominent role not only in new cell development and differentiation, but also in determining the survival of mature cells in the nervous system (Table 1). Each of forkhead transcription factors may have different biological effects during development. For example,* Foxo3a *−/− and* Foxo4 *−/− mice can develop without incidence and have similar weight gain [216]. Yet, mice singly deficient in* Foxo1* die by embryonic day eleven and lack development of the vascular system [216]. Overexpression of FoxO1, such as in skeletal muscle in mice, can lead to weight loss, reduced skeletal muscle mass, and impaired glycemic control [217]. On further analysis, FoxO3a −/− null animals experience a number of developmental abnormalities that were not present in mice singly deficient for FoxO4.* Foxo3a *−/− mice are known to become infertile with ovarian follicles that are depleted of oocytes [218]. FoxO3a overexpression retards oocyte growth and follicular development and leads to anovulation and luteinization of unruptured follicles [219], indicating a specific function for FoxO3a in the development and maintenance of the reproductive system. This work may suggest a role for FoxO3a in relation to oocyte and follicular development [220]. Mutations in* FOXO3a* and* FOXO1a* have been reported in a small percentage of women who suffer from premature ovarian failure [221]. Deletion of* Foxo1*,* Foxo3a*, and* Foxo4* or a single deletion of* Foxo3a* also blocks the repopulation of hematopoietic stem cells in murine models [186, 222], illustrating the need for FoxO proteins to maintain hematopoietic stem cells. Other work suggests that FoxO3a alone may play a role in maintaining hematopoietic stem cells, since hematopoietic stem cells are decreased in aged FoxO3 −/− mice compared to the littermate controls [222]. FoxO3 in combination with type 2 deiodinase (D2) and circulation thyroid hormone also is necessary for normal mouse myogenesis and muscle regeneration [223]. Nuclear translocation of FoxO1 in cooperation with SMAD3/4 and Sp1 by transforming growth factor *β* (TGF*β*) is required for oligodendrocyte progenitor development and myelination in the central nervous system [224].

In contrast, other studies suggest that inhibition of FoxO protein activity or prevention of Wnt pathway disruption may be necessary for stem cell survival. FoxO1 may negatively affect pancreatic beta cell survival [225]. Work that examines osteoblastogenesis demonstrates that FoxO proteins during oxidative stress and aging may antagonize Wnt signaling pathways and block the proliferation of osteoblast precursors [226]. SIRT1 deficiency in mouse embryonic stem cells has been shown to enhance the acetylation and phosphorylation FoxO1, block nuclear localization of FoxO1, and prevent apoptotic cell death that would otherwise ensue with FoxO1 activity [227]. SIRT1 is also necessary to promote cortical bone formation with osteoblast progenitors by deacetylating FoxOs and preventing FoxO protein binding to *β*-catenin and inhibiting Wnt signaling [228].

In the nervous system, FoxO proteins similarly determine the fate of neuronal precursors and the maintenance of neurons [137, 229]. Studies that employ genetic deletions of Foxa1 and Foxa2 in mice result in the decline of striatal dopamine metabolites, reduction in dopaminergic cells, and locomotor deficits [230]. Stem cell maintenance may also be governed by the interactions between WISP1 and FoxO proteins. WISP1 is upregulated during stem cell migration [231] and WISP1 may be one of several components that affect induced pluripotent stem cell reprogramming [232, 233]. WISP1 requires *β*-catenin for the differentiation of marrow derived mesenchymal stem cells [234]. During oxidative stress, FoxO may bind to *β*-catenin and prevent stem cell development similar to the previously described pathways with Wnt signaling [212, 235]. Cellular mechanisms that utilize Wnt signaling such as EPO also control FoxO protein activity for stem cell growth [236–241]. EPO promotes erythroid progenitor cell development that requires the modulation of FoxO3a activity [46, 172, 242, 243]. Other trophic factors, such as glial cell line-derived neurotrophic factor, require the inhibition of FoxO1 and FoxO3a to promote rat enteric nervous system precursor development [244].

In relation to neurodegenerative disorders and neuronal cell survival, activation of FoxO proteins under most conditions leads to cell death [13, 245]. Manganese toxicity that may be a factor in neurodegenerative disorders such as Parkinson's disease has been associated with the cell death of astrocytes through increased expression and activation of FoxO proteins [246]. Iron-induced oxidative stress that results in apoptotic death of hippocampal neurons can lead to a protective response that activates Akt and blocks FoxO protein translocation to the nucleus [55]. Protection of primary hippocampal neurons by group I metabotropic receptors during exposure to ROS requires the phosphorylation and inactivation of FoxO3a as well as the prevention of caspase cleavage of FoxO3a [102] to block the generation of potentially “proapoptotic” amino-terminal (Nt) fragments [105]. Antioxidant administration to protect cortical neurons and hippocampal neuronal cell lines during excitotoxicity [247] and in experimental models of AD with A*β* toxicity [48] employs FoxO3 inactivation and blocked translocation to the cell nucleus [247]. Independent of Wnt signaling, EPO has been shown to offer neuronal and vascular cell protection through pathways that inactivate FoxO proteins, such as FoxO3a [46, 52]. During cerebral ischemia, FoxO3a expression increases in the hippocampus [248] and FoxO3a interaction with cell cycle induction proteins may play a role in neuronal apoptotic cell death [44]. Toxin exposure in cortical neurons that fosters FoxO3a activation and p27 (kip1) transcription leads to apoptosis [249]. In microglial cells of the nervous system as well as neurons, knockdown of FoxO3a and prevention of nuclear shuttling lead to the increased survival during oxidative stress [47, 104]. During periods of elevated glucose, cortical neurons [250] and vascular cells [28, 42, 53, 58] are protected through inhibitory phosphorylation of FoxO3a and the nuclear export of this protein.

However, it is important to recognize the antiproliferative and anticancer effects of FoxO proteins that make these transcription factors attractive targets for the inhibition of tumor growth [14, 50]. Increased activity of FoxO3a with cyclin-dependent kinase inhibitor p27 in isolated human breast cancer cells can suppress breast cancer progression [251]. Colorectal cancer progression may be checked by the activation of FoxO1 [252] and angiogenesis that is necessary for tumor growth can be blocked by the activation of FoxO3a [253]. Through the disruption of proliferative pathways such as Wnt signaling, a number of cancers that include breast cancer, gastric cancer, central nervous system tumors, and lung carcinoma [190, 209, 212, 254–257] can be inhibited through FoxO protein activity [187] while loss of FoxO activity may signal an increased risk for cancer development [258]. As a result, pathways that inactivate FoxO proteins may have some potential risk for latent tumor growth.

In some experimental scenarios, FoxO protein activation may be required for neuronal protection. Blockade of neurodegenerative disease and adverse behavioral deficits during selenium exposure that may be linked to the development of amyotrophic lateral sclerosis occurs during increased FoxO protein expression [259]. FoxO3a also may be necessary for cochlear auditory activity and the maintenance of synaptic function [41]. In Drosophila models of A*β* toxicity, loss of FoxO results in decreased survival and locomotive activity [260]. FoxO proteins such as FoxO3 may also be important for the control of autophagic flux in Parkinson's disease [261]. In dopaminergic neurons, overexpressing human *α*-synuclein, inhibition of FoxO3 is protective. However, a small degree of FoxO3 activity prevents nigral neuron cell death in the presence of human *α*-synuclein accumulation by reducing the amount of *α*-synuclein and fostering the accumulation of autophagic vacuoles containing lipofuscin [261]. Interestingly, a controlled upregulation of FoxO3a and SIRT1 expression in cardiac tissue may be important during exercise [262]. Levels of SIRT1 that are less than 7.5-fold are associated with catalase expression that is also controlled by FoxO1a to possibly reduce cell injury during oxidative stress. Conversely, elevated levels of SIRT1 at 12.5-fold can result in cardiomyocyte apoptosis and decreased cardiac function [263]. Activation of FoxO proteins may also be protective during aging. Loss of FoxO3a activity leads to decreased manganese-superoxide dismutase and enhanced cell injury with aging [264]. This extension of cellular lifespan that may be provided by FoxO proteins can be dependent on the negative regulation of Akt to allow for the activation of FoxO3a [265].

## 6. Conclusions

Neurodegenerative disorders result in significant death and disability for millions of individuals throughout the world but remain for the most part with limited treatment options and palliative therapies. Forkhead transcription factors and especially those of the FoxO subgroup are increasingly being identified as potential targets for disorders of the nervous system. FoxO proteins are expressed throughout the body, but their varied expression in the nervous system suggests that specific FoxO proteins may be vital for selective cellular and biological function and may be applicable for Alzheimer's disease, Parkinson's disease, Huntington's disease, amyotrophic lateral sclerosis, and auditory neuronal disease. For example, FoxO3 may be important for auditory synaptic transmission, cerebral endothelial vascular cell survival, and erythroid cell growth. In contrast, FoxO6 may be critical for memory consolidation and emotion. FoxOs are regulated by epigenetic and posttranslational modifications that involve phosphorylation, ubiquitylation, and acetylation by cellular pathways that involve Akt, SgK, MST1, IKK, SIRT1, and Wnt signaling to control the activity and integrity of these proteins. The ability of FoxO proteins to ultimately determine cell development and survival in the nervous system during oxidative stress resides with FoxO control of the programmed cell death pathways of apoptosis and autophagy. During oxidative stress cell injury, activation of FoxO proteins often leads to apoptotic cell death that initially fosters membrane PS residue externalization and subsequent DNA degradation. FoxO activity also can disrupt proliferative pathways of Wnt signaling involving *β*-catenin to result in apoptotic cell death. Conversely, Wnt signaling that includes WISP1 can phosphorylate, limit deacetylation, and sequester FoxO proteins in the cytoplasm to block apoptotic pathways that include caspase activation. FoxO proteins can promote autophagy to preserve cell survival during oxidative stress and clear toxic proteins from the cell. Yet, under some conditions, FoxO proteins may be tied to enhanced cell survival that is independent of autophagy. These observations do not always provide crisp conclusions and suggest the presence of a complex interplay between FoxO proteins and multiple signal transduction pathways in the cell. Furthermore, the degree of FoxO activity as well as companion pathways that involve SIRT1 can significantly impact cell development and survival. Elevated FoxO or SIRT1 activity can be detrimental to cells, but a minimal level of activity that can shepherd autophagic accumulation of toxic proteins may be beneficial. Importantly, these considerations provide further insight for the targeting of FoxO in the nervous system that may involve Wnt signaling, SIRT1, and trophic factors such as EPO to block cellular injury during oxidative stress. In addition, one should be cognizant of the nonproliferative role FoxO proteins play in tumorigenesis. Inactivation of FoxO proteins could yield unexpected cell growth not only in the nervous system but also in other regions of the body. Focusing upon FoxO proteins for the consideration of new therapeutic strategies against neurodegenerative disorders that oversee early cell development as well as differentiated cellular function can offer potentially high returns for new drug development.

## Figures and Tables

**Table 1:** Forkhead box class O (FoxO) in the nervous system.

Pathway	Function
Tissue expression	FoxO proteins are expressed in all tissues of the body FoxO proteins appear to have selective expression in the nervous system that may offer insight into the biology of specific FoxO proteins FoxO proteins may be applicable for multiple neurodegenerative disorders that include Alzheimer's disease, Parkinson's disease, Huntington's disease, amyotrophic lateral sclerosis, and auditory neuronal disease

Epigenetic and posttranslational modification	FoxO proteins are controlled by posttranslation protein modifications that involve phosphorylation, acetylation, and ubiquitylation that involve Akt, SgK, MST1, IKK, SIRT1, and Wnt signaling with WISP1

Oxidative stress	FoxO proteins may be required for oxidative stress to result in apoptosis and can disrupt proliferative pathways of Wnt signaling FoxO proteins have been linked to disease progression and oxidative stress can modify FoxO interactions with other proteins that can ultimately influence cell neuronal survival

Autophagy and apoptosis	During oxidative stress, FoxO proteins can lead to the induction of autophagy and promote cell survival to clear the presence of toxic proteins such as mHtt, *α*-synuclein, and A*β* Under some circumstances with autophagy, a reduction in autophagy is required for protection indicating that FoxO cytoprotection may not always be directly tied to the induction of autophagy Protection against apoptosis usually requires inhibition or gene knockdown of FoxO proteins to protect against injuries such as cerebral ischemia, microglial and inflammatory cell demise, and A*β* exposure. Protection with metabotropic glutamate receptors, NAD^+^ precursors, and trophic factors such as EPO requires inhibition and nuclear export of FoxO proteins

Stem cells	Activity of FoxO proteins can be necessary for the development of hematopoietic stem cells, dopaminergic cells, muscle regeneration, and oligodendrocyte progenitor development and myelination At times, reduction in FoxO protein activity is required for cell development and differentiation such as with pancreatic beta cell survival, osteoblast precursors, embryonic stem cells, and enteric nervous system precursors

Akt: protein kinase B; A*β*: beta-amyloid; EPO: erythropoietin; IKK: I*κ*B kinase; MST1: mammalian sterile 20-like kinase-1; mHtt: mutant Huntingtin; SgK: NAD^+^: nicotinamide adenine dinucleotide; serum- and glucocorticoid-inducible protein kinase; SIRT1: silent mating type information regulation 2 homolog 1 (*S. cerevisiae*); WISP1: wnt1 inducible signaling pathway protein 1.
